# Overuse of antibiotics for urinary tract infections in pregnant refugees, Lebanon

**DOI:** 10.2471/BLT.23.291235

**Published:** 2024-03-27

**Authors:** Christine Al Kady, Krystel Moussally, Wafaa Chreif, Anna Farra, Severine Caluwaerts, Heiman Wertheim, Dounia Soukarieh, Fabiola Gordillo Gomez, Johanna Dibiasi, Annick Lenglet

**Affiliations:** a South Beirut Project, Lebanon Mission, Operational Center Brussels, Médecins Sans Frontières, Domtex Building, Fifth Floor, Hamra Main Street, Beirut, Lebanon.; b Middle East Medical Unit, Médecins Sans Frontières, Beirut, Lebanon.; c Medical Department, Médecins Sans Frontières, Brussels, Belgium.; d Department of Medical Microbiology, Radboud University Medical Center, Nijmegen, Kingdom of the Netherlands.; e Coordination Department, Lebanon Mission, Beirut, Lebanon.; f International Centre for Antimicrobial Resistance Solutions, Copenhagen, Denmark.

## Abstract

**Objective:**

To determine whether adding urine culture to urinary tract infection diagnosis in pregnant women from refugee camps in Lebanon reduced unnecessary antibiotic use.

**Methods:**

We conducted a prospective, cross-sectional study between April and June 2022 involving pregnant women attending a *Médecins Sans Frontières* sexual reproductive health clinic in south Beirut. Women with two positive urine dipstick tests (i.e. a suspected urinary tract infection) provided urine samples for culture. Bacterial identification and antimicrobial sensitivity testing were conducted following European Committee on Antimicrobial Susceptibility Testing guidelines. We compared the characteristics of women with positive and negative urine culture findings and we calculated the proportion of antibiotics overprescribed or inappropriately used. We also estimated the cost of adding urine culture to the diagnostic algorithm.

**Findings:**

The study included 449 pregnant women with suspected urinary tract infections: 18.0% (81/449) had positive urine culture findings. If antibiotics were administered following urine dipstick results alone, 368 women would have received antibiotics unnecessarily: an overprescription rate of 82% (368/449). If administration was based on urine culture findings plus urinary tract infection symptoms, 144 of 368 women with negative urine culture findings would have received antibiotics unnecessarily: an overprescription rate of 39.1% (144/368). The additional cost of urine culture was 0.48 euros per woman.

**Conclusion:**

A high proportion of pregnant women with suspected urinary tract infections from refugee camps unnecessarily received antibiotics. Including urine culture in diagnosis, which is affordable in Lebanon, would greatly reduce antibiotic overprescription. Similar approaches could be adopted in other regions where microbiology laboratories are accessible.

## Résumé

**Objectif:**

Déterminer si l'ajout d'une uroculture pour diagnostiquer une infection urinaire chez les femmes enceintes dans les camps de réfugiés au Liban permet d’éviter de recourir inutilement aux antibiotiques.

**Méthodes:**

Nous avons mené une étude transversale prospective entre avril et juin 2022 sur des femmes enceintes fréquentant une clinique de santé sexuelle et reproductive gérée par *Médecins Sans Frontières* dans le sud de Beyrouth. Les femmes présentant deux tests positifs par bandelette urinaire (c'est-à-dire souffrant potentiellement d'une infection urinaire) ont fourni des échantillons d'urine pour culture. Les tests d'identification bactérienne et de sensibilité aux antimicrobiens ont été effectués conformément aux lignes directrices du Comité européen des antibiogrammes. Nous avons comparé les caractéristiques des femmes ayant obtenu des résultats d'uroculture positifs et négatifs puis nous avons calculé la proportion d'antibiotiques surprescrits ou utilisés de façon inappropriée. Enfin, nous avons estimé combien coûterait l'ajout d'une uroculture à l'algorithme de diagnostic.

**Résultats:**

L'étude a porté sur 449 femmes enceintes susceptibles de souffrir d'infections urinaires: 18,0% (81/449) ont obtenu des résultats positifs lors de l'uroculture. Si des antibiotiques avaient été administrés uniquement sur la base des résultats aux tests par bandelette urinaire, 368 femmes en auraient reçu sans en avoir besoin, ce qui équivaut à un taux de surprescription de 82% (368/449). S'ils avaient été administrés sur la base des résultats de l'uroculture ainsi que des symptômes d'infection urinaire, 144 des 368 femmes ayant obtenu des résultats négatifs auraient reçu des antibiotiques sans en avoir besoin, ce qui représente un taux de surprescription de 39,1% (144/368). La mise en place de l'uroculture a engendré un coût supplémentaire de 0,48 euro par femme.

**Conclusion:**

Dans les camps de réfugiés, une proportion élevée de femmes enceintes potentiellement atteintes d'infections urinaires se sont vu prescrire des antibiotiques sans que ce ne soit nécessaire. Inclure dans le diagnostic une uroculture, proposée à prix abordable au Liban, permettrait de réduire considérablement leur surprescription. Des approches similaires pourraient en outre être adoptées dans d'autres régions où des laboratoires de microbiologie sont accessibles.

## Resumen

**Objetivo:**

Determinar si la adición del urocultivo al diagnóstico de infección urinaria en mujeres embarazadas de campos de refugiados en Líbano redujo el uso innecesario de antibióticos.

**Métodos:**

Entre abril y junio de 2022, se realizó un estudio prospectivo transversal en el que participaron mujeres embarazadas que acudían a una clínica de salud reproductiva sexual de Médicos Sin Fronteras en el sur de Beirut. Las mujeres con dos pruebas de tira reactiva en orina positivas (es decir, sospecha de infección urinaria) proporcionaron muestras de orina para cultivo. La identificación bacteriana y las pruebas de sensibilidad antimicrobiana se realizaron siguiendo las directrices del Comité Europeo sobre Pruebas de Susceptibilidad Antimicrobiana. Se compararon las características de las mujeres con urocultivos positivos y negativos, y se calculó el porcentaje de antibióticos prescritos en exceso o utilizados de forma inadecuada. También se calculó el coste de añadir el urocultivo al algoritmo diagnóstico.

**Resultados:**

En el estudio participaron 449 mujeres embarazadas con sospecha de infección urinaria: el 18,0% (81/449) tuvieron resultados positivos en el urocultivo. Si la administración de antibióticos se basaba únicamente en los resultados de la tira reactiva de orina, 368 mujeres habrían recibido antibióticos sin necesidad: una tasa de prescripción excesiva del 82% (368/449). Si la administración se basaba en los resultados del urocultivo más los síntomas de infección urinaria, 144 de las 368 mujeres con resultados negativos del urocultivo habrían recibido antibióticos sin necesidad: una tasa de sobreprescripción del 39,1% (144/368). El coste adicional del urocultivo fue de € 0,48 por mujer.

**Conclusión:**

Un alto porcentaje de embarazadas con sospecha de infección urinaria procedentes de campos de refugiados recibieron antibióticos innecesariamente. La inclusión del urocultivo en el diagnóstico, asequible en Líbano, reduciría en gran medida la prescripción excesiva de antibióticos. Se podrían adoptar enfoques similares en otras regiones donde se pueda acceder a los laboratorios de microbiología.

## ملخص

**الغرض:**

تحديد ما إذا كانت الاستعانة باختبار مزرعة البول في تشخيص أمراض المسالك البولية، لدى السيدات الحوامل من مخيمات اللاجئين في لبنان، قد أدت إلى الحد من الاستخدام غير الضروري للمضادات الحيوية.

**الطريقة:**

قمنا بإجراء دراسة استباقية متعددة القطاعات في الفترة ما بين أبريل/نيسان ويونيو/حزيران 2022، وشملت السيدات الحوامل اللاتي يترددن على عيادة الصحة الإنجابية الجنسية التابعة لمنظمة أطباء بلا حدود ( *Médecins Sans Frontières *) في جنوب بيروت. السيدات اللواتي حصلن على نتيجة إيجابية لاختبارين لشريط الغمس في البول (أي مرض مشتبه فيه في المسالك البولية)، قدمن عينات لإجراء مزرعة للبول. تم إجراء اختبار لتحديد البكتيريا، واختبار الحساسية تجاه مضادات الميكروبات، وفقًا للمبادئ التوجيهية للجنة الأوروبية لاختبار الحساسية لمضادات الميكروبات. قمنا بمقارنة خصائص السيدات اللواتي حصلن على النتائج الإيجابية والسلبية لمزرعة البول، وقمنا بحساب نسبة المضادات الحيوية التي تم وصفها بشكل زائد، أو المستخدمة بشكل غير مناسب. كما قمنا أيضًا بتقدير تكلفة الاستعانة باختبار مزرعة البول في آلية التشخيص.

**النتائج:**

شملت الدراسة 449 سيدة حامل يشتبه في إصابتهن بأمراض المسالك البولية: %18.0 (81/449) كان لديهن نتائج إيجابية لمزرعة البول. إذا تم إعطاء المضادات الحيوية بعد نتائج اختبار شريط الغمس في البول وحده، فإن 368 سيدة من الممكن أن تكون قد تلقت مضادات حيوية غير ضرورية: معدل الوصف المفرط %82 (368/449). إذا كان الإعطاء يعتمد على نتائج مزرعة البول بالإضافة إلى أعراض مرض المسالك البولية، فإن 144 من 368 سيدة ذات نتائج سلبية لمزرعة البول، من الممكن أن تكون قد تلقت مضادات حيوية غير ضرورية: معدل الوصف المفرط %39.1 (144/368). وكانت التكلفة الإضافية لمزرعة البول 0.48 يورو لكل سيدة.

**الاستنتاج:**

هناك نسبة عالية من السيدات الحوامل اللاتي يشتبه في إصابتهن بأمراض المسالك البولية من مخيمات اللاجئين، يتلقين مضادات حيوية غير ضرورية. إن الاستعانة بمزرعة البول في التشخيص، وهو أمر ميسور التكلفة في لبنان، من شأنه أن يحد بشكل كبير من الوصف المفرط للمضادات الحيوية. ويمكن بانتهاج أساليب مشابهة في مناطق أخرى حيث تتواجد مختبرات ميكروبيولوجية.

## 摘要

**目的:**

确定对黎巴嫩难民营中孕妇进行尿路感染诊断时增加尿液培养是否能够减少不必要的抗生素使用。

**方法:**

在 2022 年 4 月至 6 月期间，我们针对在贝鲁特南部的一家生殖和性健康诊所（*无国界医生组织成员*）就诊的孕妇，进行了一项前瞻性横断面研究。我们对两次尿液试纸检测均呈阳性（即疑似尿路感染）的孕妇提供的尿液样本进行了培养。并根据欧洲抗菌药物敏感性试验委员会的指南进行了细菌鉴定和抗菌药物敏感性试验。我们比较了尿液培养结果呈阳性和阴性的孕妇的特征，并计算了抗生素处方过量或不当使用的比例。我们还估算了将尿液培养加入诊断流程的成本。

**结果:**

该研究纳入了 449 名疑似尿路感染的孕妇：其中 18.0% (81/449) 的尿液培养结果呈阳性。如果仅根据尿液试纸检测结果即给予抗生素，则有 368 名孕妇将接受不必要的抗生素治疗：处方过量率为 82% (368/449)。如果根据尿液培养结果和尿路感染症状给药，则在 368 名尿液培养结果呈阴性的孕妇中将有 144 名接受不必要的抗生素治疗：处方过量率为 39.1% (144/368)。尿液培养的额外费用为每名孕妇 0.48 欧元。

**结论:**

难民营中有很大一部分疑似尿路感染的孕妇接受了不必要的抗生素治疗。将尿液培养纳入诊断流程将大大降低抗生素的处方过量率，并且在黎巴嫩，该费用是负担得起的。在其他有微生物实验室的地区也可以采用类似的方法。

## Резюме

**Цель:**

Определить, снижает ли добавление посева мочи к диагностике инфекции мочевыводящих путей у беременных женщин из лагерей беженцев в Ливане степень ненужного использования антибиотиков.

**Методы:**

В период с апреля по июнь 2022 года было проведено проспективное перекрестное исследование с участием беременных женщин, посещающих клинику сексуального и репродуктивного здоровья *«Врачи без границ» (Médecins sans frontières, MSF)* на юге Бейрута. Женщины с двумя положительными результатами анализа мочи с помощью тест-полоски (то есть с подозрением на инфекцию мочевыводящих путей) сдавали образцы мочи на посев. Идентификация бактерий и тестирование на чувствительность к антимикробным препаратам проводились в соответствии с рекомендациями Европейского комитета по тестированию чувствительности к антимикробным препаратам. Было проведено сравнение характеристик женщин с положительными и отрицательными результатами исследования культуры мочи, а также рассчитана доля излишне назначенных или неправильно использованных антибиотиков. Также была оценена стоимость добавления посева мочи в диагностический алгоритм.

**Результаты:**

В исследование были включены 449 беременных женщин с подозрением на инфекции мочевыводящих путей: у 18,0% (81/449) были положительные результаты анализа посева мочи. Если бы антибиотики назначались только по результатам анализа мочи, 368 женщин получали бы антибиотики без необходимости: частота избыточного назначения составила 82% (368/449). Если бы назначение препарата основывалось на результатах анализа мочи и симптомах инфекции мочевыводящих путей, то 144 из 368 женщин с отрицательными результатами анализа мочи получали бы антибиотики без необходимости: частота избыточного назначения составила 39,1% (144/368). Дополнительные расходы на посев мочи составили 0,48 евро на одну женщину.

**Вывод:**

Значительная часть беременных женщин с подозрением на инфекции мочевыводящих путей из лагерей беженцев получала антибиотики без необходимости. Включение в диагностику посева мочи, который в Ливане доступен по цене, значительно сократит передозировку антибиотиков. Подобные подходы могут использоваться и в других регионах, где есть доступ к лабораториям микробиологии.

## Introduction

Inappropriate antibiotic use is one of the main drivers of antimicrobial resistance.^1^ The standard of care for pregnant women is to give antibiotics to prevent complications, particularly in those with a urinary tract infection and asymptomatic bacteriuria. However, overprescription is common and previous studies have found that antibiotics were overprescribed in up to 96% of pregnant women with suspected urinary tract infections.^2^^,^^3^

In pregnant women, a urinary tract infection can present symptomatically or asymptomatically.^3^^,^^4^ If left untreated or if treated inappropriately, infection can lead to complications for mothers (e.g. pyelonephritis, septicaemia or acute respiratory distress syndrome) and can result in preterm delivery, intrauterine growth restriction or a low-birth weight infant.^5^^,^^6^ Therefore pregnant women should undergo screening for urinary tract infections to ensure that infections are accurately diagnosed and treated effectively. This approach can be very challenging during conflicts and among refugees because quality health care may be lacking, drugs may be unavailable, and timely and effective access to diagnostic services, including microbiology laboratories, may be costly and limited or nonexistent.^7^^,^^8^

International clinical guidelines recommend that pregnant women should be screened for urinary tract infections during their first antenatal care visit. For those with positive urinary tract infection screening test results, urine culture should be conducted to confirm the diagnosis, and antibiotic sensitivity testing should be performed to select the most appropriate antibiotic therapy.^6^^,^^9^^,^^10^ Rapid screening methods for urinary tract infections, such as the urine dipstick test, are often the only options available in low-resource settings and humanitarian situations but they lack reliability. A recent meta-analysis showed that urine dipstick tests had a pooled sensitivity of 73% and a specificity of 89% for detecting asymptomatic bacteriuria during pregnancy.^11^ Another meta-analysis published in 2004 concluded that urine dipsticks were effective for ruling out infection when both nitrite and leukocyte results were negative, but were not reliable as stand-alone tests to confirm the presence of a urinary tract infection.^12^

At the *Médecins Sans Frontières* (MSF) sexual reproductive health clinic in the Burj al-Barajneh refugee camp in south Beirut, Lebanon, pregnant women are screened for urinary tract infections during each antenatal care visit using a repeated CombiScreen urine dipstick test (Analyticon Biotechnologies GmbH, Lichtenfels, Germany) that can detect the presence of nitrites and leukocytes, as specified by MSF guidelines.^13^ Women who have two positive urine dipstick test results are given either a single 3 g oral dose of fosfomycin trometamol or receive 200 mg oral doses of cefixime twice daily for 5 days. No confirmatory tests are performed. 

The aim of our study was to investigate the effect of adding urine culture to the diagnostic algorithm for urinary tract infections on the prescription of antibiotics for pregnant Syrian refugees who attended an MSF antenatal care clinic in Lebanon. In addition, we determined whether the use of a repeated positive urine dipstick test result alone to diagnose urinary tract infections in these women, in accordance with the current MSF protocol, led to inappropriate antibiotic prescription and usage.

## Methods

Since 1949, more than 30 000 refugees are estimated to have settled in the overcrowded Shatila and Burj al-Barajneh camps in the southern suburbs of Beirut,^14^ where they face extremely challenging living conditions, including high unemployment, limited infrastructure and unpredictable security.^15^ The main provider of free primary health care in both camps remains MSF, which has, since 2014, offered antenatal, postnatal and family planning services through an outpatient centre that caters for a monthly average of 1300 antenatal care consultations (*Médecins Sans Frontières*, unpublished data, 2023).

We performed an analytical, cross-sectional study involving prospectively collected data on pregnant women aged 18 years or older who: (i) received routine antenatal care at an MSF clinic in south Beirut between 27 April and 30 June 2022; and (ii) had two consecutive, positive, urine dipstick test results during a single clinic visit. Each woman was included only once. We excluded women who had received antibiotics in the week before their visit, or who were eligible for urine culture according to the current MSF clinical algorithm (i.e. three consecutive, positive, urine dipstick test results at three different antenatal care visits). 

**Fig. 1:**
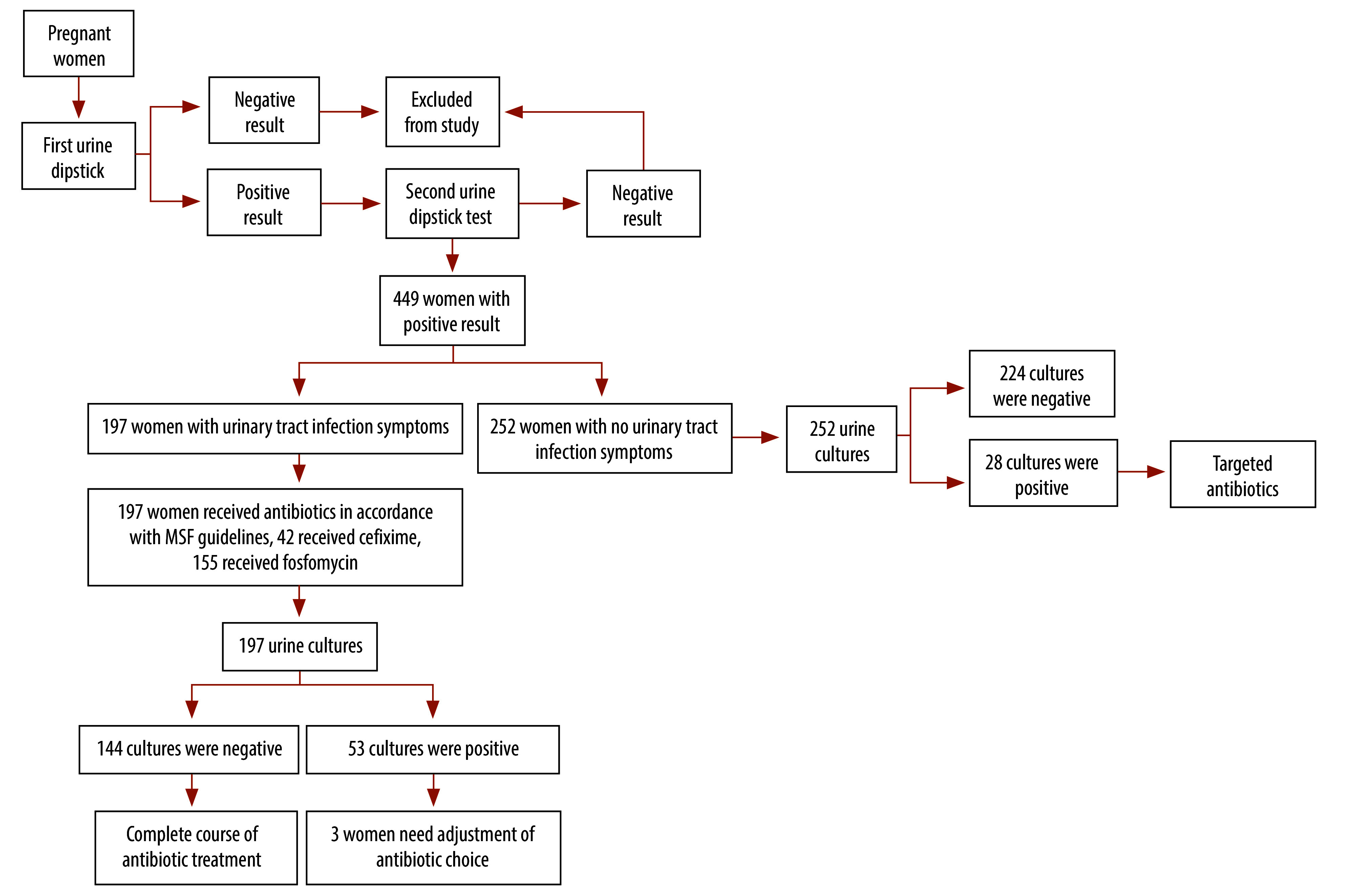
Diagnosis and treatment flowchart, study of antibiotic overuse for urinary tract infection during pregnancy, Lebanon, 2022

Each woman enrolled in the study provided two midstream urine samples on the same day. The first sample was used for the first dipstick test. If either the nitrite or leukocyte result was positive, a second urine sample was collected after precleaning with soap and water. The second sample was used for a repeat dipstick test and for urine culture. For our study, we recorded the nitrite and leucocyte results from the second positive dipstick test. In women with symptoms of a urinary tract infection, women provided both urine samples before starting antibiotic treatment (Fig. 1).

MSF: *Médecins Sans Frontières*.

Notes: Women with symptoms of a urinary tract infection were prescribed and received antibiotics after providing a urine sample for culture. Of the 28 women without urinary tract infection symptoms who had positive urine culture findings, 23 (82.1%) received fosfomycin, three (10.7%) received cefixime and two (7.1%) received the combination of amoxicillin and clavulanic acid.

If a woman reported any symptoms of sexually transmitted infection or vaginitis during the visit, she was offered an inspection of the vulva and a speculum exam checking for cervical and/or vaginal inflammation or discharge, as part of her routine care. Before examination, the patient had to give verbal consent, as per usual practice in the clinics.

Urine cultures were processed at the Saint Marc laboratory in Beirut, a microbiology laboratory whose quality had previously been validated for urine testing by MSF. The laboratory reported culture results within 48 hours. Bacteria were isolated and enumerated and bacterial growth was assessed using a cystine, lactose, electrolyte-deficient agar and 1 or 10 µL loops. Bacteria were identified primarily using a stain test (i.e. Gram staining) supplemented by tests that assessed whether the bacteria contained or had the ability to produce certain enzymes (e.g. catalase and oxidase) and an analysis of each bacterium’s characteristic colony morphology on the agar. Antibiotic susceptibility testing was interpreted according to the guidelines of the European Committee on Antimicrobial Susceptibility Testing (EUCAST; version 12.0, valid from 1 January 2022) and an antibiogram was generated for each positive urine culture to identify each organism’s susceptibility to different antimicrobials.^16^

We extracted sociodemographic and routine clinical data from electronic health records and additional data were retrieved from patients’ medical files and from a pre-drafted study questionnaire that asked about urinary tract infection history, recent antibiotic use and urinary tract infection symptoms. We obtained microbiology through an online laboratory information system.

### Data analysis

We defined a suspected urinary tract infection as two consecutive, positive, urine dipstick test results (i.e. the presence of nitrites or a leukocyte level of 2 or higher) during a single clinic visit. A confirmed urinary tract infection was defined as positive urine culture findings, as indicated by bacterial growth of at least 10 000 colony-forming units (CFUs) per mL, irrespective of the presence of clinical signs of infection. To avoid undertreatment, we used this cut-off value combined with the presence of a maximum of two uropathogens to confirm the need for urinary tract infection treatment.

We defined overprescription of antibiotics as the prescription of antibiotics for a suspected urinary tract infection when it was unnecessary, as indicated by negative urine culture findings. An inappropriate antibiotic was defined as a prescribed antibiotic whose use was not consistent with antibiotic susceptibility findings among women in the study with confirmed urinary tract infections.

To accurately assess the overprescription of antibiotics, we determined that a conservative sample of 450 pregnant women was needed. We assumed that 50% of women with two positive dipstick results on the same visit would have a confirmed urinary tract infection on urine culture, and considered a 95% confidence interval (CI) with a 5% level of precision and a 15% nonresponse rate (OpenEpi Calculator, OpenEpi). We report findings using descriptive statistics and consider a *P*-value of 0.05 or less as significant.

From the microbiology data, we calculated the proportion of women with a confirmed urinary tract infection and estimated the proportion of antibiotics overprescribed. We also identified individual bacterial species isolated from urine cultures. In addition, information from antibiotic susceptibility testing was used to estimate the proportion of antibiotics used inappropriately. Susceptibility results reported as “I – susceptible, increased exposure” were grouped with results reported as “S – susceptible, standard dosing regimen”, except for antibiotics without clinical breakpoint concentrations in their respective EUCAST categories.^16^ All analyses were performed using Stata v. 14.0 (StataCorp LLC, College Station, United States of America).

In estimating the additional cost of urine culture for suspected urinary tract infections, we considered the cost of: (i) the urine dipstick tests, urine culture tests (including urine cups) and antibiotics that would have been provided hypothetically under routine conditions for all enrolled women; and (ii) the antibiotic susceptibility tests used and antibiotics actually prescribed under research conditions for women with positive urine culture findings. We calculated the mean cost per patient with and without urine culture. Estimated costs were calculated in euros (€) and based either on local pricing in Lebanon (e.g. for urine cultures and antibiograms) or on MSF pricing (e.g. for imported urine dipsticks and antibiotics). Table 1 summarizes the costs included.

**Table 1:** Diagnosis and treatment costs, study of antibiotic overuse for urinary tract infection during pregnancy, Lebanon, 2022

Item	Cost of urinary tract infection diagnosis and treatment							
	Without urine culture			With urine culture, negative culture findings			With urine culture, positive culture findings	
	Units	Unit price (€)		Units	Unit price (€)		Units	Unit price (€)
**Urine dipstick**	2	0.10		2	0.10		2	0.10
**Urine culture**	NA	NA		2	2.01		2	2.01
**Urine cup**	2^a^	0.10		3^a^	0.10		3**^a^**	0.10
**Antibiogram test**	NA	NA		NA	NA		1	3.23
**Antibiotic**								
Fosfomycin^b^	1	3.00		NA	NA		1	3.00
Cefixime^b^	1	1.75		NA	NA		1	1.75
Other^c^	NA	NA		NA	NA		1	2.24

€: Euro; NA: not applicable.

^a^ Two urine cups (one for each dipstick) are needed in the absence of urine culture; with urine culture, an additional cup is used and sent to the laboratory for testing.

^b^ The costs shown are for full courses of each antibiotic: without urine culture, the costs were for antibiotics provided hypothetically under routine conditions for all enrolled women; with urine culture, the costs were for antibiotics actually prescribed following antibiotic susceptibility testing.

^c^ Other antibiotics were used when the causative organism was resistant to both fosfomycin and cefixime. The combination of amoxicillin and clavulanic acid was the only additional antibiotic used in the study.

The study protocol was approved by the MSF ethics review board (ID: 2155, 6 December 2021) and the Lebanese American University institutional review board (LAU.SOM.AF2, 7/Apr/2022). All women provided written informed consent.

## Results

The study included 449 pregnant women with a suspected urinary tract infection, as indicated by two consecutive, positive, urine dipstick test results during a single clinic visit (Fig. 1): their mean age was 25.5 years (standard deviation, SD: 5.9); 93.5% (420/449) were Syrian; 38.1% (171/449) were in their second trimester; 76.2% (342/449) were multigravida; and 57.9% (260/449) had had one to three live births.

Of the 449 women, 81 (18.0%) had their urinary tract infection diagnosis confirmed on urine culture, whereas 368 (82.0%) had negative urine culture findings (Fig. 1). Among the 81 with confirmed urinary tract infections, 59 (72.8%) had a uropathogen concentration greater than 10^5^ CFU/mL, whereas 22 (27.2%) had a concentration between 10^4^ and 10^5^ CFU/mL. Of the 22 with a concentration less than or equal to 10^5^ CFU/mL, 14 (63.6%) had at least one clinical symptom indicative of a urinary tract infection at the time of the visit, whereas eight (36.4%) had no symptoms. Compared with women who had negative urine culture findings, women with a confirmed urinary tract infection were significantly more likely to be in their first pregnancy or first trimester, and to have a urinary tract infection for the first time during their current pregnancy (Table 2).

**Table 2:** Characteristics of pregnant women, by urine culture findings, study of antibiotic overuse for urinary tract infection during pregnancy, Lebanon, 2022

Characteristic	Women with a suspected urinary tract infection^a^			*P* ^b^
	All (*n* = 449)	With positive urine culture findings (*n* = 81)	With negative urine culture findings (*n* = 368)	
**Sociodemographic characteristics at study enrolment**				
Age in years, mean (SD)	25.5 (5.9)	25.9 (5.9)	25.4 (5.9)	0.506
Nationality, no. (%)				
Syrian	420 (93.5)	75 (92.6)	345 (93.8)	0.701
Other	29 (6.5)	6 (7.4)	23 (6.2)	NA
Residence, no. (%)				
Inside the camp^c^	171 (38.0)	30 (37.0)	141 (38.3)	0.830
Outside the camp^c^	278 (62.0)	51 (63.0)	227 (61.7)	NA
**Pregnancy-related characteristic**				
Gravidity, no. (%)				
First pregnancy	107 (23.8)	27 (33.3)	80 (21.7)	0.028
2–5 previous pregnancies	274 (61.1)	39 (48.1)	235 (63.9)	NA
> 5 previous pregnancies	68 (15.1)	15 (18.6)	53 (14.4)	NA
Previous abortions,^d^ no. (%)				
None	290 (64.6)	50 (61.7)	240 (65.2)	0.352
1–3	147 (32.7)	27 (33.3)	120 (32.6)	NA
> 3	12 (2.7)	4 (5.0)	8 (2.2)	NA
Parity, no. (%)				
0	134 (29.8)	30 (37.0)	104 (28.3)	0.295
1–3	260 (57.9)	42 (51.9)	218 (59.2)	NA
> 3	55 (12.3)	9 (11.1)	46 (12.5)	NA
Gestational age in weeks, no. (%)				
0–13 (first trimester)	116 (25.8)	30 (37.0)	86 (23.4)	0.029
14–27 (second trimester)	171 (38.1)	29 (35.8)	142 (38.6)	NA
> 27 (third trimester)	162 (36.1)	22 (27.2)	140 (38.0)	NA
**Clinical characteristics of previous urinary tract infection in current pregnancy**				
Previous urinary tract infection in current pregnancy, no. (%)				
No	289 (64.4)	62 (76.5)	227 (61.7)	0.011
Yes	160 (35.6)	19 (23.5)	141 (38.3)	NA
Urinary tract infection treatment provider during current pregnancy, no. (%)^e^				
MSF	136 (85.0)	16 (84.2)	120 (85.1)	1.0
Other than MSF	24 (15.0)	3 (15.8)	21 (14.9)	NA
Antibiotic used to treat previous urinary tract infection, no. (%)^f^				
Fosfomycin	102 (66.2)	12 (63.2)	90 (66.7)	0.762
Cefixime	52 (33.8)	7 (36.8)	45 (33.3)	NA
**Clinical characteristics of current urinary tract infection**				
Urine dipstick results, no. (%)				
Leukocyte-positive	446 (99.3)	78 (96.3)	368 (100)	0.006
Leukocyte-negative	3 (0.7)	3 (3.7)	0 (0)	NA
Nitrite-positive	28 (6.2)	27 (33.3)	1 (0.3)	< 0.001
Nitrite-negative	421 (93.8)	54 (66.7)	367 (99.7)	NA
Urinary tract infection symptoms present, no. (%)				
Yes	197 (43.9)	53 (65.4)	144 (39.1)	< 0.001
No	252 (56.1)	28 (34.6)	224 (60.9)	NA
No. of urinary tract infection symptoms (%)				
0	252 (56.1)	28 (35.6)	224 (60.9)	< 0.001
1	90 (20.0)	25 (30.9)	65 (17.7)	NA
2	74 (16.5)	20 (24.7)	54 (14.7)	NA
3	33 (7.4)	8 (8.8)	25 (6.7)	NA
Type of urinary tract infection symptom, no. (%)^g,h,i^				
Dysuria	162 (82.2)	44 (83.0)	118 (81.9)	0.861
Pelvic pain	63 (31.9)	17 (32.1)	46 (31.9)	0.986
Urinary frequency	112 (56.8)	28 (52.8)	84 (58.3)	0.489
Antibiotic prescribed for current urinary tract infection symptoms, no. (%)				
Yes	199^j^ (44.3)	55 (67.9)	144 (39.1)	< 0.001
No	250 (55.7)	26 (32.1)	224 (60.9)	NA
**Other clinical characteristics**				
Pre-existing diabetes,^k^ no. (%)				
Yes	4 (0.9)	1 (1.2)	3 (0.8)	0.550
No	445 (99.1)	80 (98.8)	365 (99.2)	NA
Suspected STI or abnormal vaginal discharge at enrolment,^m^ no. (%)				
Yes	99 (22.1)	17 (20.9)	82 (22.3)	0.799
No	350 (77.9)	64 (79.1)	286 (77.7)	NA
Findings on exam at enrolment, no. (%)^n,o^				
Vaginitis	26 (26.3)	2 (11.8)	24 (29.3)	0.120
Cervicitis	49 (49.5)	10 (58.8)	39 (47.6)	0.648
Vaginal candidiasis	76 (76.8)	11 (64.7)	65 (79.3)	0.375

MSF: *Médecins Sans Frontières*; NA: not applicable; SD: standard deviation; STI: sexually transmitted infection.

^a^ A suspected urinary tract infection was defined as two consecutive, positive, urine dipstick test results during a single clinic visit.

^b^ *P*-values for the difference in positive and negative results were calculated using a *χ^2^* or Fisher’s exact test for categorical variables or using an unpaired *t*-test for continuous variables.

^c^ The Shatila and Burj al-Barajneh refugee camps in south Beirut.

^d^ Abortions included spontaneous and induced terminations of pregnancy.

^e^ Percentage of women with a history of urinary tract infection during the current pregnancy.

^f^ Percentages were calculated for 154 women with a history of urinary tract infection during the current pregnancy because data on antibiotic use for the previous urinary tract infection were not available for six women.

^g^ Percentage of women with current symptoms.

^h^ Each woman may have had more than one symptom.

^i^ No woman with urinary tract infection symptoms had haematuria.

^j^ The number of women prescribed an antibiotic (i.e. 199) is higher than the number with symptoms (i.e. 197) because two patients without symptoms were prescribed an antibiotic based on the medical team’s judgment before the urine culture findings were available, which deviated from the protocol.

^k^ No woman had a pre-existing immune deficiency disease, sickle cell disease or a urinary tract malformation.

^m^ Sexually transmitted infections (STIs) were suspected using a syndromic approach based on speculum observations.

^n^ Percentage of women with a diagnosed STI or abnormal vaginal discharge at enrolment.

^o^ Each woman may have had more than one diagnosis at the same visit.

At enrolment, 43.9% (197/449) of women had at least one symptom of a urinary tract infection (Fig. 1); dysuria was the most common (82.2%, 162/197). Among the 81 with a confirmed urinary tract infection, 28 (34.6%) had asymptomatic bacteriuria. Women with positive urine culture findings were significantly more likely to present with a symptom than those without: the proportions were 65.4% (53/81) and 39.1% (144/368), respectively (*P* < 0.001; Table 2). Thirty of the 144 (20.8%) women who had symptoms of a urinary tract infection but negative urine culture findings were diagnosed with a sexually transmitted infection based on speculum observations. Almost all women (96.4%, 27/28) with a positive nitrite result on their urine dipstick test had a confirmed urinary tract infection (Table 2). Nitrite results on the dipstick test had a specificity of 99.7% (367/368) and a sensitivity of 33.3% (27/81) for positive urine culture findings.

### Overprescription of antibiotics

We found that, if the urine dipstick test was the only tool used for diagnosis of a urinary tract infection (standard practice in many low- and middle-income countries and routinely used by the MSF south Beirut clinic), 368 (144 with urinary tract infection symptoms plus 224 without) of the 449 women with two positive urine dipstick test results would have received antibiotics unnecessarily because urine culture subsequently showed they did not have a urinary tract infection (Fig. 1). This finding corresponds to an overprescription of 82.0% (368/449). Moreover, 43.9% (197/449) of women enrolled in our study received empirical antibiotics before their urine culture findings were available because they presented with at least one urinary tract infection symptom: of these, 42 (21.3%) received cefixime and 155 (78.7%) received fosfomycin. However, 144 of the 197 (73.1%) subsequently had negative urine culture findings and, hence, were unnecessarily prescribed antibiotics. If only women with symptoms who had two positive urine dipstick results were given empirical antibiotic treatment (i.e. under research conditions), 144 of all 368 women with negative urine culture findings would have received antibiotics unnecessarily, which corresponds to an overprescription rate of 39.1% (144/368; Fig. 1).

### Microbiology findings

Among the 81 women with positive urine culture findings, the most frequently isolated organism was *Escherichia coli*, which was present in 64 (80.0%) cultures. The other organisms detected were *Proteus* species in nine women (11.2%), *Klebsiella pneumoniae* in seven (8.7%) and *Enterococcus faecalis* in one (1.2%). Although the presence of extended spectrum β-lactamases (ESBLs) was not assessed, we estimated that 20.0% (16/80) of the Gram-negative bacteria responsible for urinary tract infection were ESBL producers because they were resistant to third-generation cephalosporins (e.g. ceftriaxone) but susceptible to cefoxitin and β-lactamase inhibitors. Almost half of all bacterial isolates were resistant to cefixime, and one *E. coli* isolate showed fosfomycin resistance (Table 3). Cefixime would have been used inappropriately for three of 13 isolates (23.1%) that would have been treated with the drug under routine conditions, because the isolates were found to be resistant on susceptibility testing and fosfomycin would have been used inappropriately for one *E. coli* isolate.

**Table 3:** Antibiotic susceptibility of organisms isolated, study of antibiotic overuse for urinary tract infection during pregnancy, Lebanon, 2022

Antibiotic treatment	No. of isolates susceptible to antibiotic (%)			
	*E. coli* (*n* = 64)	*Proteus* spp. (*n* = 9)	*K. pneumoniae* (*n* = 7)	Total (*n* = 80)^a^
Amoxicillin and clavulanic acid	48 (75.0)	7 (77.8)	3 (42.9)	58 (72.5)
Cefixime^b^	33 (52.4)^c^	4 (44.4)	4 (66.7)^c^	41 (52.6)
Cefoxitin	59 (95.2)^c^	8 (88.9)	6 (100)^c^	73 (94.8)
Ceftriaxone	48 (75.0)	8 (88.9)	5 (71.4)	61 (76.2)
Ciprofloxacin	53 (82.8)	7 (77.8)	6 (85.7)	66 (82.5)
Fosfomycin^b,d^	63 (98.4)	NA	NA	63 (98.4)^e^
Nitrofurantoin^d^	58 (93.5)^c^	NA	NA	58 (93.5)^e^
Piperacillin and tazobactam	63 (98.4)	9 (100)	7 (100)	79 (98.7)
Trimethoprim–sulfamethoxazole combination	31 (48.4)	6 (66.7)	3 (42.9)	40 (50.0)

*E. coli: Escherichia coli*; *K. pneumoniae: Klebsiella pneumoniae*; NA: not applicable; spp: species.

^a^ The total number of isolates was actually  81; one additional organism, *Enterococcus faecalis*, is not presented because its susceptibility to the antibiotics listed was not tested.

^b^ According to *Médecins Sans Frontières’* clinical guidelines, cefixime and fosfomycin are first-line treatments for women with urinary tract infections.

^c^ Two results for cefixime (one for *E. coli* and one for *K. pneumonia*), three for cefoxitin (two for *E. coli* and one for *K. pneumonia*) and two for nitrofurantoin (both for *E. coli*) were excluded from the calculations because they were reported as “I – susceptible, increased exposure” although their clinical breakpoint concentrations were not listed in the European Committee on Antimicrobial Susceptibility Testing guidelines used.^16^

^d^ As clinical breakpoint concentrations for fosfomycin and nitrofurantoin were available only for *E. coli,* the two antibiotics were not tested on other isolates.

^e^ The denominator was the number of *E. coli* isolates only.

### Costs

The estimated total cost of diagnosis and treatment for all 449 women included in the study under routine conditions (i.e. without urine culture) was €1400 compared to €1620 with urine culture, which corresponds to a mean cost per woman of €3.12 (95% CI: 3.07–3.17) and €3.60 (95% CI: 3.40–3.80), respectively. The mean difference was €0.48 per woman. Not administering antibiotics to the 368 women with negative urine culture findings could have saved €992.70.

## Discussion

We found that less than one fifth of pregnant women who had a suspected urinary tract infection based on positive urine dipstick results alone had their infection confirmed on urine culture. Hence, adding reliable urine culture to the diagnostic algorithm would avoid most of these women being overprescribed antibiotics. We also found that, if only women with symptoms and two positive urine dipstick results received antibiotics, about two out of five women would be overprescribed antibiotics.

Promoting antimicrobial stewardship during the outpatient treatment of urinary tract infections is particularly challenging in low-resource settings due to diagnostic uncertainties, the limitations of existing guidelines and rising antimicrobial resistance.^17^ Previous studies reported antibiotic overuse rates above 96% for urinary tract infections in pregnant women in some contexts,^2^^,^^17^^–^^19^ often because treatment was based on clinical suspicion or because rapid screening methods were used rather than microbiological confirmation.^17^ In our study, around half of confirmed urinary tract infections involved a pathogen resistant to cefixime, which is a recommended first-line treatment and is categorized as a Watch antibiotic on the World Health Organization (WHO) AWaRE (Access, Watch, Reserve) list of antibiotics.^20^ This finding highlights the need: (i) to reconsider the use of urine dipsticks for diagnosing urinary tract infections to decrease the overuse of Watch antibiotics; (ii) to improve monitoring of appropriate antibiotic prescriptions with systematic point-prevalence surveys; and (iii) to propose alternative treatments for use in Lebanon, such as nitrofurantoin – an Access antibiotic to which *E. coli* is highly susceptible. Although nitrofurantoin is suggested to be a safe and reasonable first-line treatment for urinary tract infections in pregnancy,^21^ concerns exist about the risk of congenital malformation in the first trimester and about neonatal haemolytic anaemia near delivery.^22^ Nitrofurantoin remains an option to consider, but implementing a tailored prescribing algorithm for each trimester in humanitarian settings might be challenging.

The levels of antibiotic overuse and inappropriate use we found in this study contribute to antimicrobial resistance, which is particularly concerning during pregnancy because antibiotic-resistant bacteria may be transmitted to neonates and cause adverse neonatal outcomes.^23^^,^^24^ Therefore, ensuring that antibiotics are used rationally and appropriately during pregnancy is a must, particularly given the increase in antimicrobial resistance both globally and among Syrian refugees.^25^^,^^26^

Our estimate that 20% of organisms found in our study participants produced ESBLs may have been too low as previous studies have reported higher proportions: for example, 32% in urinary tract infection cases in Lebanon and 52% in urine samples in the Syrian Arab Republic.^27^^,^^28^ However, the proportion may have been low in our study because the women included were young on average and very few had a history of diabetes. Risk factors for the acquisition of ESBL-producing bacteria by pregnant women and the general population include: (i) prior antibiotic use; (ii) recurrent urinary tract infections within the past year; (iii) prior hospitalization; (iv) diabetes mellitus; and (v) older age (as observed in pregnant Syrian women).^28^^–^^34^

We found that nitrite positivity on the urine dipstick test was a reliable indicator of a confirmed urinary tract infection, as has previously been observed for Gram-negative pathogens, mostly Enterobacterales.^35^^–^^38^ However, a negative nitrite result does not exclude the possibility of positive urine culture findings; in particular, the relationship between the two may be influenced by urinary tract infection epidemiology.^38^ In Lebanon, if urine culture cannot be added to the diagnostic algorithm, the overprescription of antibiotics in pregnant women with urinary tract infections could possibly be reduced by requiring either a positive nitrite result on the urine dipstick test or the presence of two or more urinary tract infection symptoms. This approach should be considered cautiously as urine dipstick tests for leukocytes and nitrites are less accurate than urine culture.^39^ According to WHO recommendations, in settings where there is limited access to urine culture, on-site Gram staining of midstream urine samples is preferred for diagnosing asymptomatic bacteriuria in pregnant women unless financially unviable.^40^ In this situation, dipstick tests should be considered despite the higher likelihood of a false-positive result than with Gram staining.^40^ Our results were consistent with previous reports that *E. coli, Proteus* species and *Klebsiella* species isolates from samples (including urine) collected in the Syrian Arab Republic were resistant to cefixime.^41^ Antibiotic resistance profiles may vary over time as the epidemiology of antimicrobial resistance evolves. Therefore, considering local changes in microbiological findings is important when choosing antibiotics to treat urinary tract infections in pregnancy.^42^

We found that adding urine culture slightly increased the average cost of urinary tract infection diagnosis and treatment. However, the difference of € 0.48 per woman we observed is unlikely to be a substantial barrier to accessing urine culture in Lebanon. In settings where urine culture is costly, not easily accessible or time-consuming, it is essential to acknowledge its utility and to explore the possibility of incorporating it into an algorithm tailored to the specific context. This approach can help reduce antibiotic overprescription. Although no diagnostic algorithm can accurately detect and appropriately treat all cases of urinary tract infection, adapting the algorithm to the context can help mitigate the negative impact of overprescription and inappropriate antibiotic use.

Our estimate of the prevalence of urinary tract infections in pregnant women may have been higher than the actual prevalence in the wider community of pregnant women because our study included only women who had a suspected urinary tract infection based on two positive urine dipstick test results. Furthermore, as our study included only women with positive urine dipstick test results, we could not calculate the overall sensitivity and specificity of the dipstick test used. Moreover, the sensitivity and specificity of the nitrite results for identifying a confirmed urinary tract infection were calculated only for women who had two positive urine dipstick test results. In addition, as our definition of a positive urine culture included a low uropathogen concentration (i.e. 10^4^ to 10^5^ CFU/mL), a quarter of infections detected and treated as urinary tract infections may have been due to skin flora contamination. Despite these limitations, we believe the overprescription of antibiotics under routine conditions is higher than the proportion we observed. Although the quality of the urine dipstick readings may have affected the accuracy of our results, we are confident that nurse training and the validation processes in place were sufficient to address uncertain dipstick readings. The costs we considered were based primarily on the price of MSF supplies – there may be slight local variations. No conclusions could be drawn about the cost–effectiveness of adding urine culture to the diagnostic process as this was outside of scope of the study.

Here we show that confirmatory urine culture was available, affordable and of a high quality. Therefore cultures should be incorporated into the diagnostic algorithm. We found that it was important to match urinary tract infection diagnostics and treatment to the local bacterial epidemiology. Such approach could be extrapolated to other settings to improve antimicrobial stewardship and the quality of care, and to help decrease the overall burden of antimicrobial resistance.
